# The Random Plots Graph Generation Model for Studying Systems with Unknown Connection Structures

**DOI:** 10.3390/e24020297

**Published:** 2022-02-20

**Authors:** Evgeny Ivanko, Mikhail Chernoskutov

**Affiliations:** 1 Institute of Mathematics and Mechanics of the Ural Branch of the Russian Academy of Sciences, 620990 Ekaterinburg, Russia; mach@imm.uran.ru; 2 Institute of Natural Sciences and Mathematics of the Ural Federal University 620075 Ekaterinburg, Russia

**Keywords:** random graph, network, omplex system, degree sequence, degree distribution

## Abstract

We consider the problem of modeling complex systems where little or nothing is known about the structure of the connections between the elements. In particular, when such systems are to be modeled by graphs, it is unclear what vertex degree distributions these graphs should have. We propose that, instead of attempting to guess the appropriate degree distribution for a poorly understood system, one should model the system via a set of sample graphs whose degree distributions cover a representative range of possibilities and account for a variety of possible connection structures. To construct such a representative set of graphs, we propose a new random graph generator, Random Plots, in which we (1) generate a diversified set of vertex degree distributions and (2) target a graph generator at each of the constructed distributions, one-by-one, to obtain the ensemble of graphs. To assess the diversity of the resulting ensembles, we (1) substantialize the vague notion of diversity in a graph ensemble as the diversity of the numeral characteristics of the graphs within this ensemble and (2) compare such formalized diversity for the proposed model with that of three other common models (Erdős–Rényi–Gilbert (ERG), scale-free, and small-world). Computational experiments show that, in most cases, our approach produces more diverse sets of graphs compared with the three other models, including the entropy-maximizing ERG. The corresponding Python code is available at GitHub.

## 1. Introduction

The random graph is a useful concept that is frequently applied in complex systems modeling. The employment of random graph models has grown rapidly with the explosive development of computer and social networks.

There is no single approach to what random graphs are or how they can be generated. If there are any characteristic properties that one can expect from the structure of the studied complex system, it may be possible to either pick a suitable random graph model (e.g., Erdős–Rényi–Gilbert, small-world, or scale-free, see Section 2.1) or to construct an bespoke model based on the maximum entropy graph model (see Section 2.2)

However, if the prototype system to be modeled is very complex and/or weakly studied, the researcher may not know its connection structure. The researcher may not even wish to spend resources on the clarification of this connection structure, as the purpose of the study may be nothing but fast prototyping.

There are many important domains where the necessary empirical dataset is too large to work with or too hard to obtain; some examples are information flows in global computer networks [1,2], large-scale social networks [3,4], signal flows in the brain [5,6], interactions of cells within a multicellular organism [7,8], and protein–protein interaction networks [9].

Furthermore, the empirical data that does exist in these domains can be ambiguous, forcing the community to regularly review the models used for them [10,11,12,13,14,15]. Finally, there are examples of complex systems (albeit rather controversial ones) where the connection structure is entirely unknown [16,17].

In the present paper, we address the question of how to construct a random graph model for a complex system, the connection structure of which cannot be (or is not intended to be) estimated empirically. As the examples in the Appendix show, real-world complex systems give rise to graphs with a huge variety of possible vertex degree distributions. This means that if a model is selected based on a guessed degree distribution (on account of a hypothesis that may turn out to be wrong), substantial errors may result; consider, for example, the scale-free graph (the first figure) as a model of either the DBLP citation database or the cell connections in a stem of *Arabidopsis Columbia* (see Table A1 in Appendix A).

In situations where information is meager, we propose that, instead of attempting to guess the true connection structure, one should model the complex system via an ensemble of graphs whose vertex degree distributions are as diverse as possible. Such an approach may prevent a narrowing in the variety of behavior that the prototype system is able to exhibit. For example, it can help to avoid “false calm” conclusions about the possible outcomes of a dangerous process run in the complex system (see Section 6.8).

As a method of constructing a diverse ensemble of graphs, we present the Random Plots model. This model produces each sample graph with *N* vertices by (1) generating a “random” nondecreasing function from [1,N] to [1,N] as a reference (we call it reference *vertex degree plot* or rVDP) or (2) applying any graph generator that implements the soft configuration model [18] to construct a graph whose array of vertex degrees sorted in ascending order (we call this the actual vertex degree plot or aVDP, see the first figure) is close to the constructed rVDP.

In the next section, we consider several common random graph models, placing emphasis on the models that represent complex systems for which no additional knowledge is available. Algorithms implementing the Random Plots model are given in Section 3. In Section 4, we consider the time complexity, space complexity, and scalability of the proposed algorithms. In Section 5, we study the accuracy of the construction, i.e., how close the aVDPs of the constructed graphs are to the corresponding rVDPs. In Section 6, we compare the diversity (in terms of several different scalar properties) of a set of graphs produced by the Random Plots model to the diversity of sets produced by the Erdős–Rényi–Gilbert, scale-free, and small-world models. We find that, with respect to six out of the seven scalar properties studied, as well as with respect to the terminal states of one dynamic process, our model produces the most diverse set of graphs. Section 7 contains some concluding remarks.

## 2. Background

In this section, we briefly outline three of the best-known models for random graph generation and consider a general approach to the construction of random graphs that meet given formalized constraints. To the best of our knowledge, the first of these models, Erdős–Rényi–Gilbert, is the only practical approach employed to represent complex systems without additional information about the connection structure. In the second part of the section, we highlight the idea that the considered theoretical approach to the construction of random graph ensembles—the maximum entropy model—also converges to the Erdős–Rényi–Gilbert model in cases where there are no constraints imposed on the graphs.

### 2.1. Three Common Random Graph Models

The Erdős–Rényi–Gilbert model

Historically, the first-developed and most-studied methods of generating random graphs were the model of Erdős and Rényi [19] and the closely related model of Gilbert [20]. In Gilbert’s approach, which is the more flexible and convenient in practice, each possible edge belongs to the graph and has a given constant probability. The vertex degrees in the resulting graph follow a Poisson distribution. Despite its intuitiveness, this model rarely captures the behavior of real-world complex systems.

The scale-free model

A much more widespread model of real-world networks is the scale-free model, where the distribution of vertex degrees follows a power law. Although there are various methods of constructing scale-free graphs [21,22,23], that of Barabási and Albert [24] remains one of the most common. The Barabási–Albert method is based on the *preferential attachment* scheme: (a) each new vertex is connected to the growing graph by one edge; (b) the probability of a new vertex connecting to one of the existing vertices of the graph is directly proportional to the degree of the latter vertex. Scale-free graphs are used as models of such complex networks as the Internet [25], the World Wide Web [26], financial networks [27], and protein–protein interaction networks [28], although there are ongoing debates about the application of the scale-free model in almost all of these examples.

The small-world model

The third model we consider is the small-world model, which describes graphs typical of social networks [29,30]. Small-world graphs combine two main properties: (a) given that two vertices are both adjacent to a third vertex, there is a high probability that they are adjacent to each other; (b) the average number of edges in the shortest path connecting an arbitrary pair of vertices is small (but greater than one). The most common way to construct small-world graphs is via the Watts–Strogatz model [31], which proceeds in two stages: (1) make a regular ring lattice such that each vertex $v_{i}$ is adjacent to the *k* vertices $v_{j}$ with the closest indices (|i−j|mod(n−k/2)≤k/2); (2) with some predefined probability, change one of the vertices of each edge to a random vertex.

**Figure 1:**
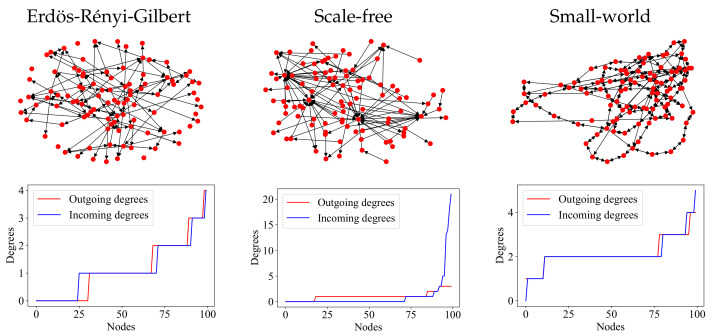
Examples of 100-vertex random graphs (upper row) and the corresponding degree distributions (lower row) constructed using different generation models: left, Erdős–Rényi–Gilbert [19]; center, scale-free [21]; right, small-world [31]. Vertex degree plots (VDPs): OY gives the vertex degree (here, both incoming and outgoing degrees are shown, but a VDP can also show just one or the other); OX gives the vertex counting number in the list ordered by ascending Y-value.

Figure 1 shows examples of graphs constructed according to the above three models. Note that the Erdős–Rényi–Gilbert graph, as a result of the “maximally stochastic” process of its construction, does not show any kind of regularity. The scale-free graph contains both the so-called *hubs* (vertices of large degree) and many leaf nodes (vertices of degree one). The small-world graph shows high connectivity among neighboring vertices, few isolated vertices, and no hubs.

The vertex degree distribution (VDD) of each graph in Figure 1 is displayed in a *vertex degree plot* (VDP). This is a convenient way to visualize the vertex degree sequence of a graph, with the vertices listed along the *X* axis in ascending order of degree and the corresponding degrees plotted along the *Y* axis. Since the VDP and the VDD of a graph contain the same information, we use the two concepts interchangeably throughout this paper.

The three models discussed above do not, of course, exhaust the vast variety of graph structures that arise in practice. Table A1 gives several real-world examples of graphs and their VDPs, some fitting within known models and others not. The variety of connection structures arising in practice, for which appropriate models are needed, is constantly expanding; for example, the recent paper [10] exhibits real-world graphs having exponential, log-normal, Weibull, and power-law-with-cutoff VDDs.

### 2.2. Theoretical Approach: Maximum Entropy Random Graph Model

In practice, when a complex system is to be modeled by a graph and some empirical information about the system is available, it is often possible to choose (or construct) a random graph model that produces sample graphs whose connection structure reflects the available empirical information [32,33,34]. The *Maximum-Entropy Random Graph Model* (MERGM) [35,36,37] allows the construction of a probability distribution with the maximum Shannon entropy on graphs that meet certain constraints expressing empirical information about the system:

(1) $$\begin{array}{cc} -\sum_{G\in G_{n}} & P(G)\log P(G)\to \max, \end{array}$$

(2) $$\begin{array}{cc} \sum_{G\in G_{n}} & P(G)=1, \end{array}$$

(3) $$\begin{array}{cc} \sum_{G\in G_{n}} & P\left(G\right){\tilde{\Omega }}_{i}\left(G\right)=\omega_{i}\in R^{k_{1}},\;i\in \bar{1,L_{1}}, \end{array}$$

(4) $$\begin{array}{cc} \; & {\bar{\Omega }}_{i}\left(G\right)=\omega_{i}\in R^{k_{2}},\;i\in \bar{1,L_{2}}, \end{array}$$

where $G_{n}$ is the set of graphs with *n* vertices (can be any type of graph, e.g., simple digraphs); ${\bar{\Omega }}_{i}$ are the properties that should be met exactly by each generated graph (e.g., in the case of a labeled graph, the number of edges equals ω), and ${\tilde{\Omega }}_{j}$ represents the properties that should be met on average (e.g., the average degree of the vertex $v_{i}$ over the graphs of the resulting ensemble should equal $\omega_{i}$). The MERGM with the latter constraint is sometimes referred to as *the Soft configuration model* [18]. A heuristic algorithm addressing this problem is used as part of the random graph model proposed in this paper.

The global optimization problem (1)–(4) can be solved with standard methods, e.g., Lagrange multipliers [36]. It should be noted that expressing the available information about the modeled complex system in the form of constraints (3) and (4) and obtaining a probability distribution as the solution to (1)–(4) does not necessarily mean that a neat algorithm that produces random graphs in accordance with the obtained distribution can be easily constructed. Consider, for example, an $\tilde{\Omega }\left(G\right)$ of the form “the process Ξ being run on *G* ends in one of its terminal states according to some predefined probability distribution”. Depending on the complexity of process Ξ, the generating algorithm can be no more elegant than brute-force Monte-Carlo sampling.

In this work, we are interested in complex systems, the information about which is meager, so we confine ourselves to only two assumptions: (1) the possible number *n* of elements in the system is bounded, $n_{min}\leq n\leq n_{max}$ and (2) a simple unlabeled digraph (directed graph with indistinguishable vertices without loops and multiple edges) is a suitable model for the system. The first assumption automatically limits the number *m* of connections in the simple digraphs, 0≤m≤n(n−1). Whereas the first assumption seems rather natural (one is unlikely to face a discrete complex system for which it is impossible to set *any* limits on the number of elements), the second one needs some explanation. When choosing between labeled vs. unlabeled as well as directed vs. undirected graphs, we opted for the variants that better suit the declared uncertainty about the modeled complex system. If we know almost nothing about a complex system, it is safe to assume that we also do not know its labeling. If we do not know whether the connections between the elements are symmetrical or not, we have to assume the latter as a more general case. Finally, we chose simple graphs over multigraphs because (1) the former are much more widespread and (2) simple graphs are a more challenging case in the area of graph synthesis (see the paragraph before Algorithm 2 in Section 3).

Based solely on these two assumptions, a random graph model of the studied complex system can be formulated as follows: (1) take *n* and *m* from the discrete uniform distributions $n∼U\{n_{min},n_{max}\}$ and m∼U{0,n(n−1)}; (2) use MERGM to generate graphs $G_{n,m}$.

When additional information about the prototype complex system is absent, MERGM (1)–(4) becomes (1), (2) plus either (4) in the form Edges(G)=m or (3) in the form E[Edges(G)]=m. Using the method of Lagrange multipliers, it is easy to prove (see, e.g., [36,38]) that the solution to both of these variants is the uniform distribution on $G_{n,m}$, which corresponds to the Erdős–Rényi model G(n,m). For simple *labeled* digraphs, where the edges can be uniquely identified, the probability of selection of each digraph in G(n,m) is 1/n(n−1)m.

A similar but more constructive and practically convenient approach to the construction of random graph ensembles is the Gilbert model [20] G(n,p) (with *n* vertices and with the probability of each edge existing being equal to *p*). On the labeled digraphs with *m* edges, the Gilbert and Erdős–Rényi models work identically: (1) the Gilbert model produces each particular labeled digraph $G_{n,m}$ (with *n* vertices and *m* edges) with the same probability $p^{m}{\left(1-p\right)}^{n(n-1)-m}$; (2) the probability that the Gilbert model results in *some* digraph with exactly *m* edges is n(n−1)mpm(1−p)n(n−1)−m, thus

PG(n,p)=Gn,m|Edges(G(n,p))=m=pm(1−p)n(n−1)−mn(n−1)mpm(1−p)n(n−1)−m=1n(n−1)m.

Thus, as $pn^{2}\to \infty$, by the law of large numbers, the Gilbert model converges to produce graphs with the number of edges being close to m=n(n−1)p, approaching the entropy-maximizing Erdős–Rényi model.

For *unlabeled* graphs (those with indistinguishable vertices), two different edge selections could correspond to the same graph (up to isomorphism), which can potentially skew the uniform distributions resulting from the Erdős–Rényi and Gilbert models. Fortunately, unlabeled digraphs with non-trivial automorphisms are rare. To show this, first, let us note that *undirected* unlabeled graphs are known to be rare, even for n≥40 [39,40]. Returning to unlabeled *digraphs*, note that (1) if ${\vec{G}}_{1}$ and ${\vec{G}}_{2}$ are isomorphic, then the corresponding unlabeled *graphs*$G_{1}$ and $G_{2}$ (where the undirected edge {a,b} exists in $G_{i}$ iff either (a,b) or (b,a) exists in ${\vec{G}}_{i}$) are also isomorphic; (2) the graphs $G_{i}$ constructed in this way are equivalence classes with respect to the digraphs ${\vec{G}}_{j}$. From these two notions, it follows that the share of *directed* graphs with non-trivial automorphisms does not exceed that for *undirected* graphs.

**Figure 2:**
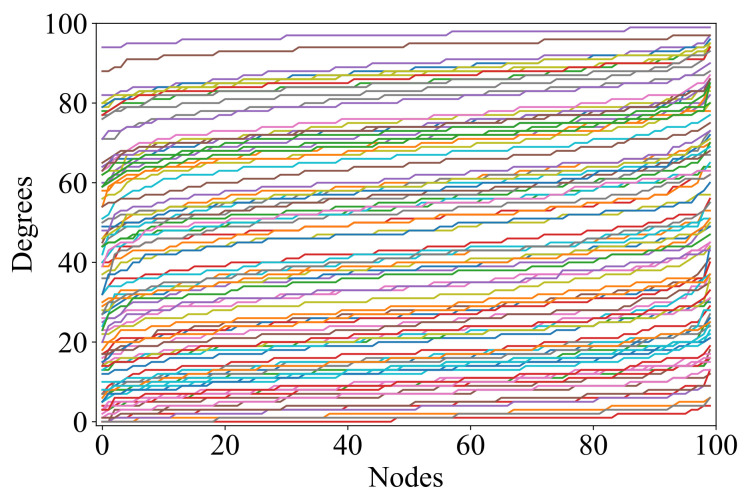
VDPs of 100 Gilbert random graphs G(n,p), where n=100,p∼U(0,1). OY gives the vertex degree; OX gives the vertex counting number in the list ordered by ascending Y-values.

The arguments given in this section suggest that the Gilbert model is a theoretically reasonable and practically feasible approach to the generation of random digraph ensembles with the maximum possible entropy, given that no additional constraints on the resulting digraphs are imposed. However, how “stochastic” are these ensembles? Consider the VDP-“portrait” of an ensemble of 100 graphs shown in Figure 2: the same shape of all VDPs in the ensemble hardly fits the intuitive understanding of stochasticity. From a practical point of view, such distribution uniformity may limit the variety of graph properties in the resulting ensembles.

## 3. Algorithms

Before proceeding to the algorithms, let us briefly recall some terms and notations that are used below. Digraph means directed graph; the indegree of a vertex in a digraph is the number of edges incoming to this vertex; and the outdegree is the the number of edges outgoing from this vertex. The notation $\bar{M,N}$ for any natural M,N:M≤N means M,M+1,M+2,…,N. The vertex degree plot (VDP) of a graph is the array of vertex degrees sorted in ascending order. A digraph has two VDPs: for the indegrees and outdegrees. The notation ξ∼U(a,b) means that the random variable ξ has a uniform distribution within the interval [a,b]. The notation ξ∼U{a,b} means *discrete* uniform distribution within $\bar{a,b}$

In the Random Plots model, the construction of each sample graph involves the consecutive execution of two algorithms: (1) an algorithm that produces a “random” (in some sense) pair of vertex in- and outdegree plots, termed *reference* VDPs (rVDPs); (2) an algorithm that generates a simple digraph whose *actual* VDPs (or aVDPs) are close to the reference ones.

Algorithm 1 produces a pair of functions (arrays) $D^{-},D^{+}:\bar{1,N}\to \bar{1,N-1}$ whose values are the target vertex in- and outdegrees of the simple digraph with *N* vertices, which is to be generated by Algorithm 2. Each array is sorted in ascending order, so that index *i* in $D^{-}\left(i\right)$ usually does not refer to the same vertex as in $D^{+}\left(i\right)$ (see examples in the ninth figure).

**Figure 3:**
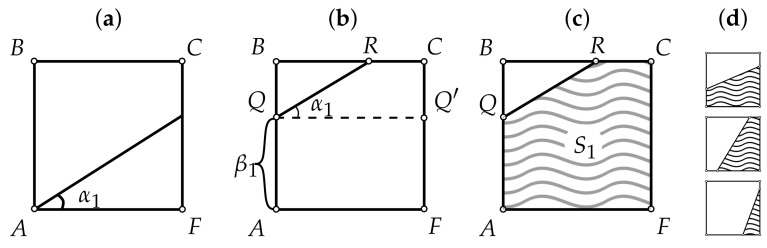
Construction of the first of two reference vertex degree plots (rVDPs) (this may be for either incoming or outgoing degrees, depending on a random choice). (**a**) Choose an angle. (**b**) Choose a shift (direction and size). (**c**) The area $S_{1}$ under the rVDP polygonal line QRC approximately shows the total number of incoming (as well as outgoing) vertex degrees in the prospective graph. (**d**) Possible forms of the rVDP depending on the particular intersection of the segment QR with the edges of ABCF, where A=(0,0),B=(0,N),C=(N,N),F=(N,0).

**Figure 4:**
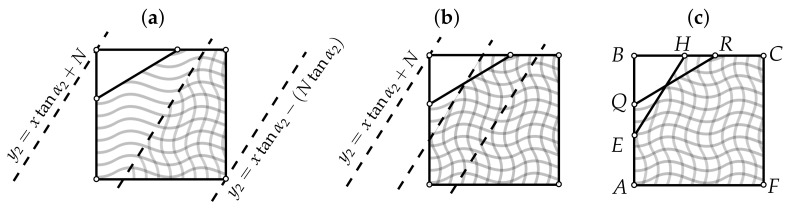
Bisection method for finding the shift $\beta_{2}$ that adjusts the area *S* under the as yet unknown outdegree reference vertex degree plots (rVDP, vertical waves) to equal the area under the already-constructed indegree rVDP (horizontal waves). (**a**) The first bisection of the initial interval with the lower bound of $\beta_{2}=-N\tan\alpha_{2}$, corresponding to the minimum S=0, and the upper bound of $\beta_{2}=N$, corresponding to the maximum $S=N^{2}$. (**b**) The second bisection. (**c**) The final step, where S(AQRCF)≈S(AEHCF).

Note that the construction of a “feasible” pair of in- and outdegree sequences, i.e., one that can, in fact, be realized by a simple digraph, is a rather difficult problem (see, for example, [41] and the comments preceding Algorithm 2 below). However, in Algorithm 1, we do not care about this kind of feasibility; the only restriction we impose on the two sequences comes from the natural equality between the sums of the outgoing and incoming degrees in a graph (see Step 8).
**Algorithm 1:** Construction of reference vertex in- and outdegree plots (rVDPs).   Let *N* be the number of elements within the complex system under study.   Let A=(0,0), B=(0,N), C=(N,N), F=(N,0) (see Figure 3a); uniformly choose an angle $\alpha_{1}∼U\left(0,\pi /2\right)$.   Choose equiprobably one of the two directions of shift: from *A* to *B* or from *A* to *F*. Without loss of generality, let it be the first direction (see Figure 3b).   Uniformly choose the shift δ∼U(0,|AB|); the equation of the line going through *Q* at an angle $\alpha_{1}$ to the horizontal is $y_{1}\left(x\right)=x\tan\alpha_{1}+\beta_{1}$, where $\beta_{1}=\delta$ (had the shift been done towards *F*, the constant term $\beta_{1}$ would have been equal to $-\delta \tan\alpha_{1}$). Given that $\alpha_{1}\in \left[0,\pi /2\right]$, *R* belongs to $\left[BC\right]\cup \left[CQ^{\prime }\right]$, so its coordinates can be easily derived (see Figure 3b). Knowing the coordinates of *R*, one can compute the area $S_{1}$ of the polygon AQRCF (see Figure 3c); this area will be approximately equal to the total number of incoming edges in the graph under construction. The other possible forms of the incoming VDP are shown in Figure 3d.   Uniformly choose an angle $\alpha_{2}∼U\left(0,\pi /2\right)$.   Choose $\beta_{2}\in R$ so that the resulting area $S_{2}$ is equal to $S_{1}$ (this corresponds to the condition of equality between the sums of the incoming and outgoing degrees). The unknown $\beta_{2}$ can be expressed analytically, but because there are many possible configurations for the intersections of the lines $y_{1}\left(x\right)=\alpha_{1}x+\beta_{1}$ and $y_{2}\left(x\right)=\alpha_{2}x+\beta_{2}$ with the edges of the square ABCF (4 variants for each line, resulting in $4^{2}$ variants for the pair $\left(y_{1},y_{2}\right)$), it may be more convenient to use the bisection method starting from the interval $\beta \in [-N\tan\alpha_{2},N]$ (see Figure 4).   Generate a random binary number b. If b=0, construct the arrays $D^{-},D^{+}$ based on the functions $y_{1},y_{2}$ and the bounding edges of ABCF so that for every $i\in \bar{1,N}$,
(5)$$\begin{array}{cc} D^{-}\left(i\right) & =Integer(\max\left\{\min\left\{y_{1}\left(i\right)+0.5,N-1\right\},1\right\}),\\ D^{+}\left(i\right) & =Integer(\max\left\{\min\left\{y_{2}\left(i\right)+0.5,N-1\right\},1\right\}), \end{array}$$
(Here, we require $D^{-}\left(i\right)\geq 1,D^{+}\left(i\right)\geq 1$, but this is an arbitrary condition that can be relaxed to $D^{-}\left(i\right)\geq 0,D^{+}\left(i\right)\geq 0$ without a loss of generality.) If b=1, use $y_{1}$ in the construction of $D^{+}$ and $y_{2}$ in the construction of $D^{-}$ instead.   If
(6)$$\sum_{i=1}^{N}D^{-}\left(i\right)=\sum_{i=1}^{N}D^{+}\left(i\right),$$
return the constructed rVDPs $D^{-},D^{+}$.   In case the sums are not equal, say
$$\sum_{i=1}^{N}D^{-}\left(i\right)>\sum_{i=1}^{N}D^{+}\left(i\right),$$
iteratively apply one of the following procedures (selected equiprobably) until (6) is true: for a random k∼U{1,N}, either (a) add 1 to $D^{+}\left(k\right)$, given that $D^{+}\left(k\right)+1\leq N-1$, or (b) subtract 1 from $D^{-}\left(k\right)$, given that $D^{-}\left(k\right)-1\geq 1$.   Return the constructed rVDPs $D^{-},D^{+}$.

**Figure 5:**
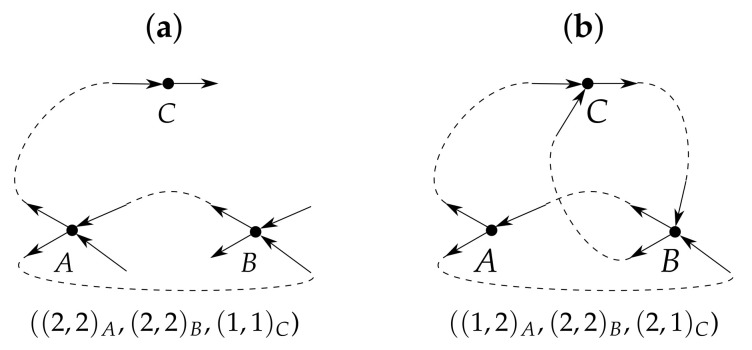
Example showing that the uncoupling of the sequence of incoming degrees from the sequence of outgoing degrees (shuffling) can result in a pair of sequences for which there exists a corresponding graph. (**a**) Given that the incident edges for *A* and *C* are already constructed so as to preserve the simplicity of the digraph, the incident edges for *B* would have to include either a loop or a multiple edge to *A*. (**b**) Changing the order of the second components makes it possible to construct a simple digraph.

Let us proceed to the second algorithm, which constructs a simple digraph whose aVDPs are close to $D^{-},D^{+}$. As we mentioned above, far from every sequence of the form

(7) $$\left(a_{1},\ldots ,a_{N}\right),\left(b_{1},\ldots ,b_{N}\right)\;\mathrm{such}\;\mathrm{that}\;\sum_{i=1}^{N}a_{i}=\sum_{i=1}^{N}b_{i},$$

allows the construction of a corresponding simple digraph, where the in- and outdegrees of the *i*th vertex are $a_{i}$ and $b_{i}$, respectively (see example in Figure 5a).

Fortunately, for our purposes, there is no need to tie the values of the outdegrees to the values of the indegrees: it is sufficient to match the degrees to the sequences $D^{-}$ and $D^{+}$ separately. Formally, the relaxed problem may be written as follows: given a sequence of the form (7), construct a simple digraph with *N* vertices for which

(8) $$\begin{array}{c} \exists \gamma :\bar{1,N}↔\bar{1,N}:\forall i\in \bar{1,N},\\ deg^{-}\left(v_{i}\right)=a_{i},\;deg^{+}\left(v_{i}\right)=b_{\gamma (i)}, \end{array}$$

where $deg^{-}\left(v_{i}\right)$ and $deg^{+}\left(v_{i}\right)$ are the in- and outdegrees of the vertex $v_{i}$ respectively.

**Figure 6:**
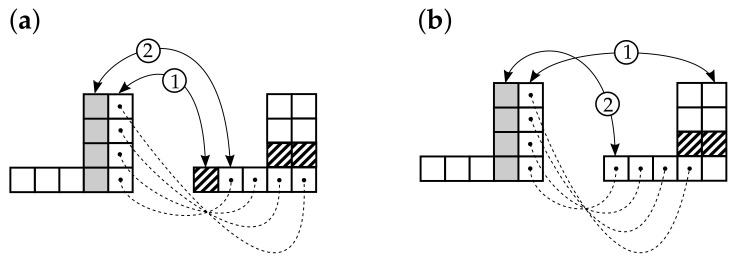
Example showing that not every pair of in- and outdegree sequences satisfying (8) can be realized by a graph, even if we pick the indegree and outdegree independently for each vertex (i.e., even if we are free to apply any permutation γ in (8)). Let the graph contain five vertices, and let the indegree and outdegree sequences both equal (1,1,1,4,4). If a vertex has outdegree 4 and indegree 1 (arrow (1) in part (**a**)), then (i) the only possible destinations for the outgoing edges from this vertex (preserving graph simplicity) are shown by the dashed lines; (ii) the outgoing edges of the second vertex of outdegree 4 (gray column) do not have enough available destinations, whether this second vertex has indegree 1, as shown by arrow (2) (the available destinations are crosshatched), or indegree 4 (this case is not shown). If the first vertex of outdegree 4 has indegree 4 (part (**b**)), the situation is similar.

While the decoupling of the indegrees from the outdegrees enlarges the set of sequences for which a digraph can be constructed (Figure 5b), it cannot guarantee the existence of a digraph for every possible pair of sequences (see example in Figure 6). It would be interesting to mathematically characterize the “weakly graphical sequences”: those for which there is at least one solution to (8).

In our case, the theoretical difficulties considered above are of no particular importance, as we are satisfied with constructing graphs whose aVDPs match only approximately the rVDPs generated by Algorithm 1. There are many works devoted to practical methods for constructing simple digraphs that match given degree sequences (at least approximately). The approach we use originated from the “sequential algorithm” of [42]; it was also influenced by earlier methods [43,44,45,46,47].
**Algorithm 2:** Construction of a digraph whose aVDPs are close to given rVDPs.Let the algorithm’s input consist of two functions $D^{-},D^{+}:\bar{1,N}\to \bar{1,N-1}$ which are the results ofAlgorithm 1. (Recall that, by construction, $D^{-}\left(i\right)\leq D^{-}\left(i+1\right)$ and $D^{+}\left(i\right)\leq D^{+}\left(i+1\right)$ for every $i\in \bar{1,N-1}$.)Let
$$D≜\sum_{i=1}^{N}D^{-}\left(i\right)\;\left(=\sum_{i=1}^{N}D^{+}\left(i\right)\;\mathrm{by}\;\mathrm{construction}\right).$$Choose an arbitrary permutation $\sigma :\bar{1,N}↔\bar{1,N}$; we are going to construct a simple digraph $G=(V≜\left\{v_{1},\ldots ,v_{N}\right\},E)$ where $deg^{-}\left(v_{i}\right)\approx D^{-}\left(i\right)$ and $deg^{+}\left(v_{i}\right)\approx D^{+}\left(\sigma \left(i\right)\right)$. (The permutation σ is used to destroy the positive correlation between $D^{-}\left(i\right)$ and $D^{+}\left(i\right)$ caused by the fact that $D^{-}$ and $D^{+}$ aremonotonically increasing.)Initially, let $deg^{-}\left(v\right)=deg^{+}\left(v\right)=0$ for all v∈V (i.e., E=⌀).While |E|<D, repeat
(a)Choose one of the two symbols + and − equiprobably; without loss of generality, let it be −,which leads to the processing of the outdegrees first and indegrees second. ( If + is chosen, then the processing order reverses with the corresponding changes in the next steps.)(b)Let $V_{-}^{\prime }$ be the set of vertices from which it is possible to start a new non-multiple edge: $V_{-}^{\prime }=\left\{v^{\prime }\in V:\exists v^{\prime \prime }\in V\backslash \left\{v^{\prime }\right\}:\left(v^{\prime },v^{\prime \prime }\right)\notin E\right\}$ ($V_{-}^{\prime }\neq ⌀$, because  if there is no pair $\left(v^{\prime },v^{\prime \prime }\right)\in V^{2}$such that $v^{\prime \prime }\neq v^{\prime }$ and $(v^{\prime },v^{\prime \prime })\notin E$, then G=(V,E) is already complete and the condition |E|<Dcould not have been true).(c)Choose a head vertex $v^{\prime }\in V_{-}^{\prime }$ with a probability of $p\left\{v^{\prime }=v_{i}\right\}=D^{-}\left(i\right)/\sum_{v_{j}\in V_{-}^{\prime }}$$D^{-}\left(j\right)$.(d)By construction $V^{\prime \prime }≜\left\{v\in V:v\neq v^{\prime }\;\&\;\left(v^{\prime },v\right)\notin E\right\}$ is not empty, choose a tail vertex $v^{\prime \prime }\in V^{\prime \prime }$ with a probability of $p\left\{v^{\prime \prime }=v_{i}\right\}=D^{+}\left(\sigma \left(i\right)\right)/\sum_{v_{j}\in V^{\prime \prime }}D^{+}\left(\sigma \left(j\right)\right)$.(e)Add $\left(v^{\prime },v^{\prime \prime }\right)$ to *E*.
Return the digraph (V,E).

Given that an rVDP is a piecewise linear approximation of the subsequent aVDP, it becomes clear why the order of degree introduced on the vertices in rVDP is important: it makes it possible to construct an approximation of reasonable accuracy using only a few line segments.

## 4. Complexity and Scalability

Algorithm 1: time O(N), space O(N)

**Figure 7:**
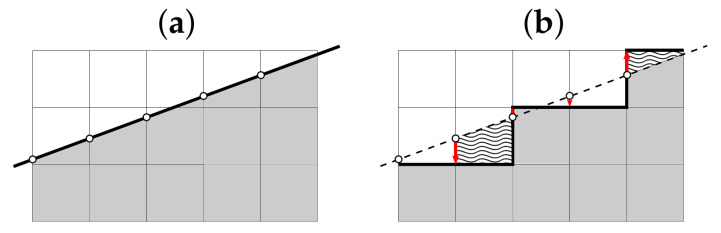
(**a**) Reference function $y_{1}$ (or $y_{2}$); (**b**) the corresponding rVDP $D^{-}$ (or $D^{+}$). The areas covered by waves show two examples of unit round-off deviations of the rVDP from its reference line at two vertices. All squares are units, so at each vertex, the deviation (waved area) cannot exceed 1; thus, the summary deviation does not exceed *N*.

Steps 1–6 of Algorithm 1 have constant time complexity O(1), Steps 7, 8 have time complexity O(N). In Step 9, we iteratively adjust possibly different $D^{-}$ and $D^{+}$. Let us estimate the number of iterations needed. Figure 7 illustrates that

$$\left|\sum_{i=1}^{N-1}\left(D^{-}\left(i\right)\;\cdot \;1\right)-\int_{1}^{N}y_{1}\left(x\right)dx\right|\leq N\;\;\;\mathrm{and}\;\;\;\left|\sum_{i=1}^{N-1}\left(D^{+}\left(i\right)\;\cdot \;1\right)-\int_{1}^{N}y_{2}\left(x\right)dx\right|\leq N.$$

Given that, by construction,

$$\int_{1}^{N}y_{1}\left(x\right)dx=\int_{1}^{N}y_{2}\left(x\right)dx,$$

we have

$$\begin{array}{c} \left|\sum_{i=1}^{N}D^{-}\left(i\right)-\sum_{i=1}^{N}D^{+}\left(i\right)\right|=\left|\left(\sum_{i=1}^{N-1}D^{-}\left(i\right)-\sum_{i=1}^{N-1}D^{+}\left(i\right)\right)+\left(D^{-}\left(N\right)-D^{+}\left(N\right)\right)\right|\\ \leq \left|\left(\sum_{i=1}^{N-1}D^{-}\left(i\right)-\int_{1}^{N}y_{1}\left(x\right)dx\right)-\left(\sum_{i=1}^{N-1}D^{+}\left(i\right)-\int_{1}^{N}y_{2}\left(x\right)dx\right)\right|\\ +\left|(D^{-}\left(N\right)-D^{+}\left(N\right))\right|\leq 2N+N, \end{array}$$

which proves that both Step 9 and, therefore, the whole Algorithm 1 have time complexity O(N).

The space complexity of Algorithm 1 is also O(N), as the largest data we need to store during the runtime consist of the *N* values of $D^{-}$ and *N* values of $D^{+}$.

Algorithm 2: time $O(N^{4})$, space $O(N^{2})$

The time complexity is O(N) for both the summation at Step 1 and permutation at Step 2. The time complexity of Step 3 is O(N). Step 4 is repeated D times at the most, where D≤N(N−1) (at each iteration we add one new edge). Step 4a is O(1), Steps 4b,c,d each have time complexity of O(N) (given that we store the lists of in- and out-neighbors for each vertex). Step 4e is O(1). Thus, the resulting time complexity of both Step 4 and Algorithm 2 as a whole is $O(N^{3})$.

The space complexity of Algorithm 2 is $O(N^{2})$, since we need to store the constructed graph and some auxiliary 1-dimensional lists, the lengths of which do not exceed *N* for each.

**Figure 8:**
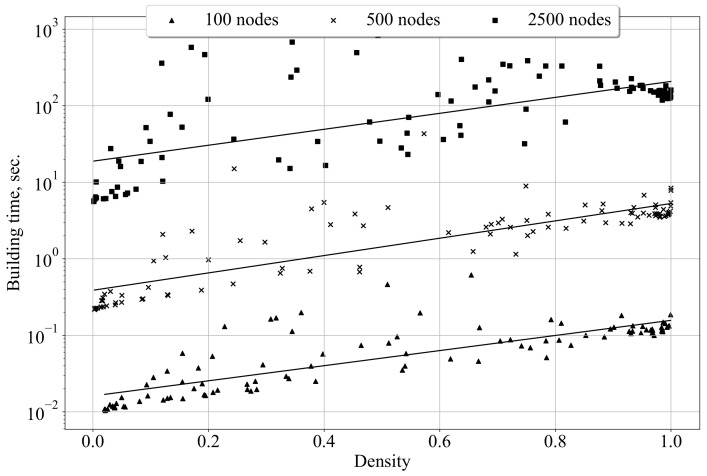
Dependence of the Random Plots graph construction time on the graph’s size and density.

The actual scalability of the combination of Algorithms 1 and 2 is shown in Figure 8. The computational experiments presented here and below were conducted using a PC with an Intel Core i7-7500U processor and 8 GB RAM. Although the time complexity of Algorithm 2 (in the worst case) is rather high–$O(N^{4})$, the experiments show that the Random Plots approach may be applied for the construction of ensembles of small- and medium-size random digraphs. To be applicable for generating ensembles of large graphs, the proposed method needs refinement.

## 5. Accuracy

In this section, we present and discuss the results of computational experiments aimed at estimating the accuracy of the aVDPs of the digraphs produced by Algorithm 2 with respect to the input rVDPs $D^{-},D^{+}$.

To evaluate how close the aVDPs of the digraphs produced by Algorithm 2 are to the corresponding rVDPs from Algorithm 1, we use the $L^{1}$ norms

(9) $$\begin{array}{c} L^{-}=\frac{1}{N(N-1)}\sum_{i=1}^{N}\left|deg^{-}\left(v_{i}\right)-D^{-}\left(i\right)\right|,\\ L^{+}=\frac{1}{N(N-1)}\sum_{i=1}^{N}\left|deg^{+}\left(v_{i}\right)-D^{+}\left(\sigma \left(i\right)\right)\right|, \end{array}$$

where *N* is the number of vertices, $D^{-}$ and $D^{+}$ are the rVDPs produced by Algorithm 1, $deg^{-}\left(v_{i}\right)$ and $deg^{+}\left(v_{i}\right)$ are the in- and outdegrees of vertex $v_{i}$ in the digraph produced by Algorithm 2, and $\sigma :\bar{1,N}↔\bar{1,N}$ is the shuffling permutation from Step 2 of Algorithm 2.

Recall that the form of the rVDPs is defined by the parameters $\alpha_{1},\beta_{1},\alpha_{2},\beta_{2}$, where $\alpha_{1}$, $\beta_{1}$, and $\alpha_{2}$ are random (see Steps 2–5 of Algorithm 1) and $\beta_{2}$ is computed uniquely (see Step 6 of Algorithm 1) to satisfy the equality (6).

**Figure 9:**
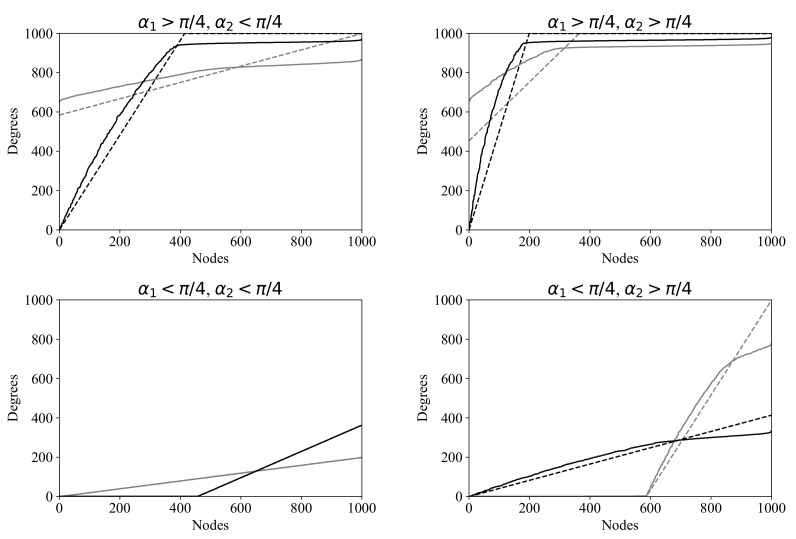
Examples of rVDPs $D^{-},D^{+}$ resulting from Algorithm 1 (dashed lines) and the corresponding aVDPs resulting from Algorithm 2 (solid lines) for graphs with 1000 vertices, for different values of $\alpha_{1}$ and $\alpha_{2}$, under the condition $\delta =0\;(\beta_{1}=0)$ (see Step 4 of Algorithm 1). Black and gray correspond to vertex in- and outdegrees, respectively. The X-value is the vertex counting number, where the vertices are listed in increasing order of degree. (In- and outdegree lists are ordered separately, so that, as a rule, each vertex corresponds to different X-values in the black and gray plots.) The Y-value is the indegree (black) or outdegree (gray).

Figure 9 illustrates the differences between the rVDPs $D^{-},D^{+}$ and the corresponding aVDPs for several distinct choices of $\alpha_{1},\alpha_{2}$ ($\beta_{1}$ is taken to be 0). Separate plots are shown depending on whether $\alpha_{1}$ and $\alpha_{2}$ are greater or less than π/4. One can see that the error grows as the plot approaches the upper bound. This is due to the following “smoothing effect”: (1) during the graph construction process, it becomes more and more difficult to find new neighbors for a high-degree vertex, as the number of vertices already connected to it increases; (2) for such vertices, Step 4b in Algorithm 2 and the subsequent resampling at Step 4a occur more and more often, pushing the new edges to less filled vertices; (3) as a result, the vertices with lower degrees in the rVDPs have greater degrees in the aVDPs, and vice versa. The smoothing effect is most pronounced at the steepest segments of the rVDP, where the plot approaches the ceiling of N−1 neighbors.

**Figure 10:**
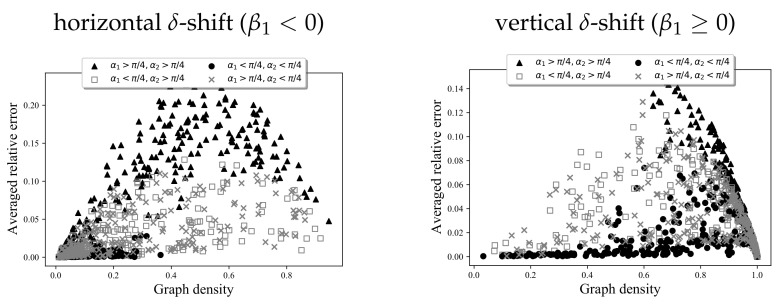
Dependence of graph construction accuracy on the graph density for different construction parameter values. Each plot contains 1000 marks. Each mark corresponds to a graph constructed by Algorithms 1 and 2, where $\alpha_{1}$, $\alpha_{2}$, and $\beta_{1}$ are selected uniformly, and $\beta_{2}$ is deterministic (see Steps 2–6 of Algorithm 1). The X-value is the graph density D/(N(N−1)) (see Step 1 of Algorithm 2); the Y-value is the averaged relative error $(L^{+}+L^{-})/2$ (see (9)).

Let us study the relationship between the coefficients $\alpha_{1},\alpha_{2},\beta_{1}$ and the accuracy of the approximation in more detail. Figure 10 shows the dependence between the “steepness coefficients” $\alpha_{1}$ and $\alpha_{2}$ and the accuracy. The low-density graphs may be clearly divided into three types: low-steepness with higher accuracy (black circles), intermediate-steepness with average accuracy (gray squares and crosses), and high-steepness with lower accuracy (black triangles). The high-density graphs follow a similar, but messier, pattern. The peak error occurs at the intermediate values of graph density, which is unsurprising, since the structural variety of graphs with intermediate density is the highest, and the more variety there is, the more chances there are for the aVDPs to deviate from the rVDPs.

**Figure 11:**
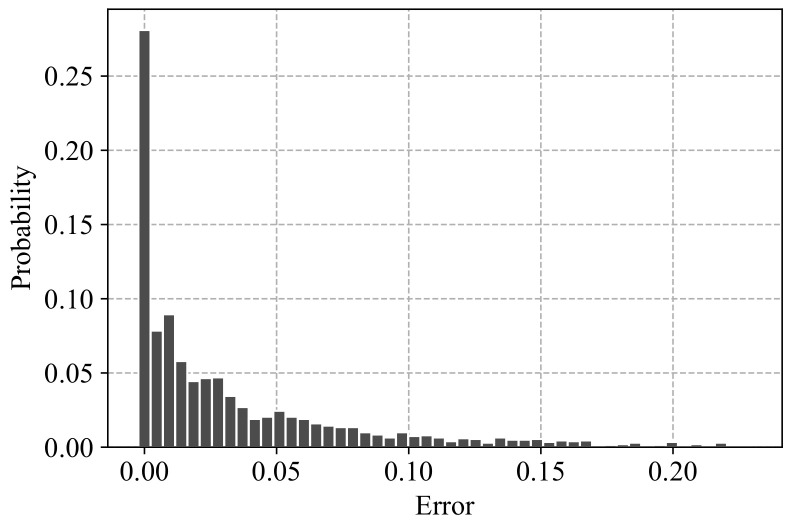
Distribution of the construction errors for the 2000 graphs used in Figure 10. The X-value is the averaged error $(L^{-}+L^{+})/2$ (see (9)); the Y-value is the probability of getting this error when applying Algorithm 2.

We conclude our analysis of the accuracy of Algorithm 2 with Figure 11, which shows the distribution of the averaged relative errors (9) for the 2000 graph generation experiments considered in Figure 10.

## 6. Coverage

As discussed in the introduction, the main motivation for this paper is to maximize the diversity of the random graphs produced. The diversity of a set of graphs is a rather abstract notion. Our approach is to associate it with the diversity of the rVDPs and to construct the latter randomly in a certain sense. However, how effective is this approach?

**Figure 12:**
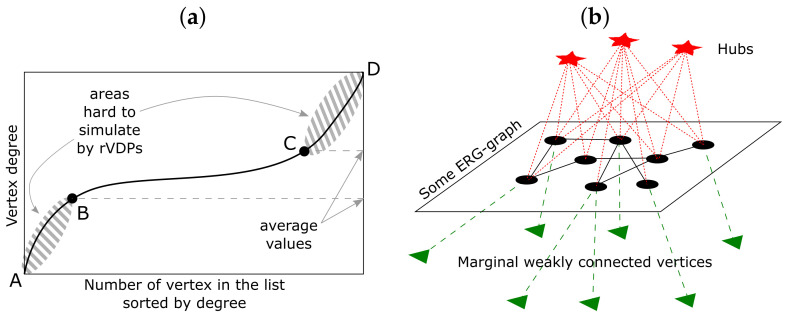
(**a**) An example of vertex degree plot that cannot be accurately simulated with the rVDPs produced by Algorithm 1; the hinge points B and C have average y-values; even the presence of one pattern of the two—either AB or CD—poses a problem for Algorithm 1 when aiming to produce a similar rVDP; (**b**) a possible graph structure that has an VDP of the form from (**a**): the marginal vertices correspond to AB, the ERG-graph–to BC, the hubs–to CD.

First, let us see how expressive the method proposed in Algorithm 1 is, i.e., how closely the rVDPs produced by Algorithm 1 can simulate some real VDPs. The last column in Table A1 shows that the most problematic VDPs have hinge points with average y-values (see Figure 12). Probably the strongest effect this issue has on the rVDPs corresponding to SF graphs: Algorithm 1 is able to produce steeply concave rVDPs (if point *Q* shifts towards *F* (see Figure 3), then according to (5), the vertex degrees on the segment [A,Q] all are set to 1), but this is the only type of concave rVDP that Algorithm 1 can produce. Note that for VDPs with a hinge point whose y-value is close to either 1 or *N*, Algorithm 1 is able to pick similar rVDPs (see, e.g., the fifth row in Table A1).

The weakness outlined above can be overcome through the use of more sophisticated forms of rVDPs generated in Algorithm 1. For example, linear $y_{1},y_{2}$ in Algorithm 1 can be replaced by piecewise linear functions with one or more breakpoints. Now, let us proceed to the practical assessment of the Random Plots method.

One natural way to measure its effectiveness is to proceed from the abstract notion of the diversity of a set of graphs to the tangible notion of the diversity of the specific scalar properties of the graphs. That is, if a set of graphs claims to be “diverse”, the graphs belonging to it should at least have diverse scalar properties.

In this section, we compare graph sets generated by our Random Plots (RP) model with sets generated by the three models described in the introduction—the Erdős–Rényi–Gilbert (ERG), scale-free (SF), and small-world (SW) models—in terms of seven well-known scalar properties of digraphs. The selection of these properties is contingent on two factors: (1) each property should be, to the greatest extent possible, typical of *directed* graphs; (2) each property should be applicable to the graphs produced by all four models considered (for example, we exclude properties defined only for weakly/strongly connected or acyclic digraphs).

In our experiments, we use each of the four models to generate 1000 digraphs on a set of 100 vertices. In each experiment, we work with a 2D parameter space where the first coordinate is the digraph density (|E|/(|V|(|V|−1))) and the second is the scalar property under consideration; each constructed graph defines a point in this parameter space. Since the proposed RP model is intended to produce maximally diverse random graphs, the cloud of 1000 points it generates in each of the 2D parameter spaces is expected to not only embrace the corresponding 1000-point clouds of the three reference models but also to cover some new areas of the parameter space.

The ERG and SF graphs are constructed using the corresponding Python 3 NetworkX 2.4 functions [48,49], while the SW graphs are constructed by [50], which allows edge directions. The ERG graph generator takes the probability $p_{e}$ for an edge to exist as a parameter (this value is the same for all the edges); in our experiments, we draw the value of $p_{e}$ for each graph from the uniform distribution U(0,1). The SW graph generator takes the probability of rewiring, β, as a parameter, which, again, is chosen uniformly: β∼U(0,1). The SF graph generator takes three basic parameters:

α—the probability of adding a new node connected by an outgoing edge to an existing node chosen randomly according to the indegree distribution; β—probability of adding an edge between two existing nodes, where one existing node is chosen randomly according [to] the indegree distribution, and the other is chosen randomly according to the outdegree distribution; and γ—the probability of adding a new node connected by an incoming edge to an existing node chosen randomly according to the outdegree distribution.

[49]

The SF graph generator requires α+β+γ=1, so we take $\alpha^{\prime },\beta^{\prime },\gamma^{\prime }∼U\left(0,1\right)$ and normalize: $\alpha =\alpha^{\prime }/\left(\alpha^{\prime }+\beta^{\prime }+\gamma^{\prime }\right)$, $\beta =\beta^{\prime }/\left(\alpha^{\prime }+\beta^{\prime }+\gamma^{\prime }\right)$, $\gamma =\gamma^{\prime }/\left(\alpha^{\prime }+\beta^{\prime }+\gamma^{\prime }\right)$.

To compute the seven scalar graph properties, we applied ready-made Python 3 NetworkX 2.4 functions wherever possible. If there were no appropriate ones, we wrote our own code. The whole project for the computational experiments is written in Python 3 and is available at [51].

Section 6.1, Section 6.2, Section 6.3, Section 6.4, Section 6.5, Section 6.6 and Section 6.7 below describe the seven scalar properties studied and summarize the results of each experiment.

### 6.1. Average Reachability Coefficient

One of the basic characteristics of graphs as models of complex distributed systems is how tightly their elements are connected to each other. There are many ways to measure this; let us start with a rather simple and straightforward one, the *average reachability coefficient*. This is computed as follows: (1) for each vertex v∈V, find the proportion of the vertices that are reachable from *v* via a (directed) path in the graph G=(V,E); (2) average this proportion over all the vertices v∈V.

**Figure 13:**
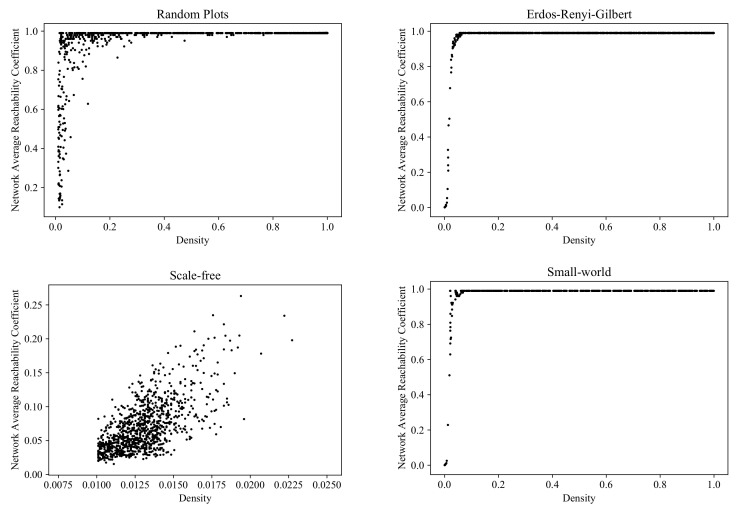
Clouds of digraphs in the plane *Density* × *Average reachability coefficient*. Each cloud consists of 1000 points. Each point corresponds to a 100-vertex graph produced by one of the four models being compared (note that the SF model does not allow the construction of dense graphs).

Figure 13 shows the clouds generated by the RP, ERG, SF, and SW models in the parameter space *Density* × *Average reachability coefficient*.

### 6.2. Size of the Largest Strongly Connected Component

**Figure 14:**
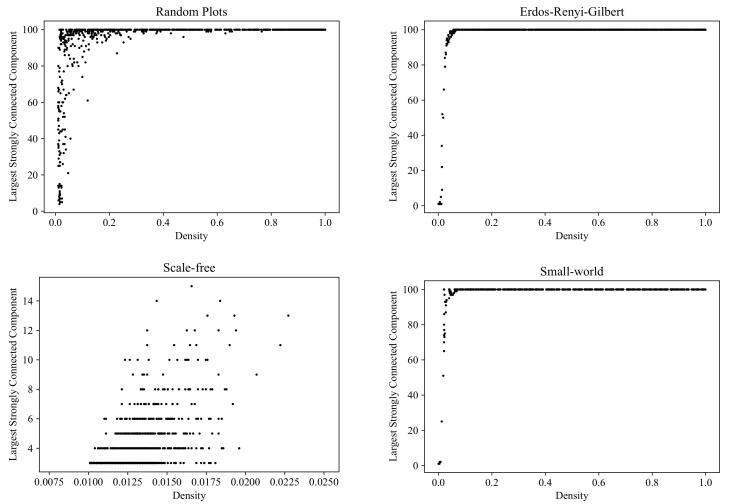
Clouds of digraphs in the plane *Density* × *Size of the largest strongly connected component*. Each cloud consists of 1000 points. Each point corresponds to a 100-vertex graph produced by one of the four models being compared (note that the SF model does not allow the construction of dense graphs).

Within a complex system, communities in which every member can reach all the other members are of particular practical interest. In digraph terms, such communities form *strongly connected components*. In Figure 14, we compare the graph sets generated by the RP, ERG, SF, and SW models in terms of the diversity of the size of the largest strongly connected component (computed with the NetworkX function [52]).

### 6.3. Spectral Bipartivity

A structure that is, in some sense, the opposite of a strongly connected component is a *part* of a bipartite graph: a part is a subset of vertices among which there are no connections.

Bipartite graphs are ubiquitous in graph theory; they are especially relevant to applications concerned with the modeling of interactions between two types of objects. In more or less homogeneous networks, bipartivity is often an unwanted property, indicating an absence of connections between the elements within some large groups and suggesting network misdesign [53].

It is known that a graph is bipartite if and only if it contains no odd cycles. This criterion suggests a refinement of the yes/no notion of bipartivity to a kind of *bipartivity measure*, given by the ratio of the number of even-length closed walks to the total number of closed walks in the network [53].

**Figure 15:**
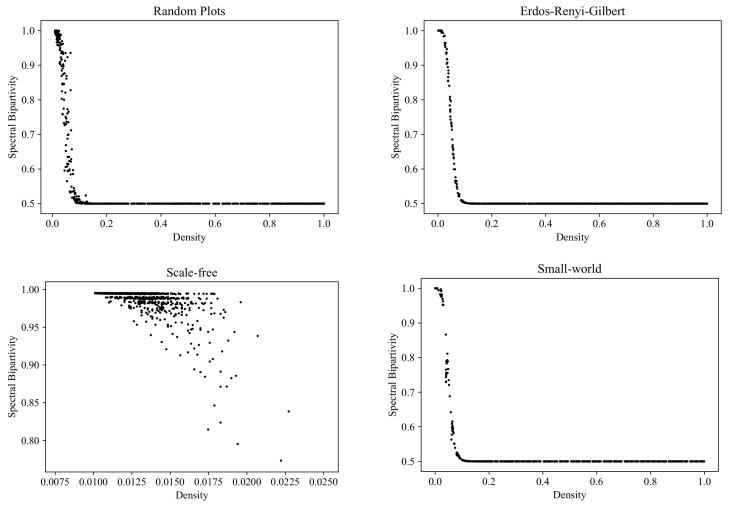
Clouds of digraphs in the plane *Density* × *Spectral bipartivity*. Each cloud consists of 1000 points. Each point corresponds to a 100-vertex graph produced by one of the four models being compared (note that the SF model does not allow the construction of dense graphs).

Figure 15 shows the RP, ERG, SF, and SW clouds corresponding to bipartivity (computed with the NetworkX function [54]).

### 6.4. Reciprocity

Now, we proceed from the properties characterizing global structures in graphs to the properties of local vertex neighborhoods. One of the basic local connectivity properties is *reciprocity* (also known as symmetry, in the context of binary relations in mathematics). The reciprocity of a digraph (V,E) is the proportion of edges for which there exists a reverse edge:

(10) |{(u,v)∈E:(v,u)∈E}|/|E|.

**Figure 16:**
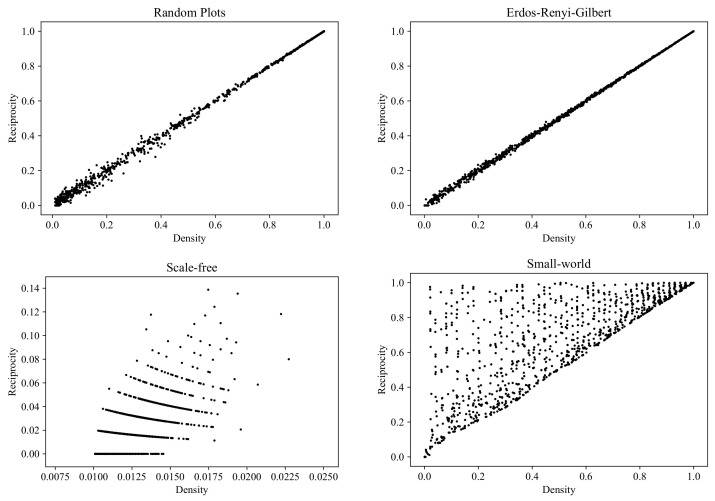
Clouds of digraphs in the plane *Density* × *Reciprocity*. Each cloud consists of 1000 points. Each point corresponds to a 100-vertex graph produced by one of the four models being compared (note that the SF model does not allow the construction of dense graphs).

Reciprocity is the only test that our RP model fails to pass. As can be seen in Figure 16, the SW model shows a much greater diversity in reciprocity values, for the following simple reason. By construction (see [55]), the SW model initially generates a regular graph with all edges being reciprocal. If the rewiring probability β is equal to 0, then all of the edges remain reciprocal and the reciprocity of the graph is equal to one, independently of the density. If β=1, the graph turns into an ERG-type graph with the corresponding typical reciprocity values (see the upper right plot in Figure 16). Since β is sampled uniformly from (0,1), the SW model produces all possible intermediate values of reciprocity for any graph density.

### 6.5. Transitivity

Another basic local connectivity property (as well as another property relevant to binary relations in mathematics) is *transitivity*. By the *degree of transitivity* of a labeled digraph (V,E) we can understand the proportion of the vertex triples for which the graph edges define a transitive relation:

$$\begin{array}{cc} V_{▵}≜ & \{\left(u,v,w\right)\in V^{3}:\left(u\neq v\neq w\neq u\right)\;\;\\ & \;\;\&\;(u,v)\in E\;\&\;(v,w)\in E\;\&\;(u,w)\in E\},\\ V_{∠}≜ & \{\left(u,v,w\right)\in V^{3}:\left(u\neq v\neq w\neq u\right)\;\;\\ & \;\;\;\;(u,v)\in E\;\&\;(v,w)\in E\}, \end{array}$$

(11) $$T_{r}=|V_{▵}\left|/\right|V_{∠}|.$$

**Figure 17:**
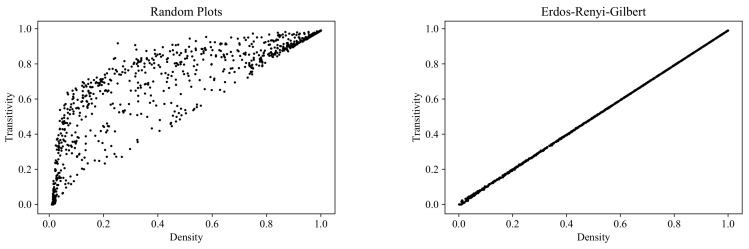

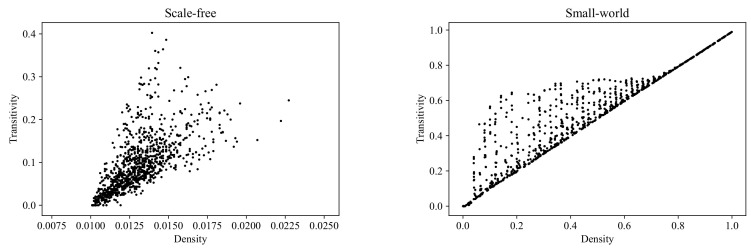
Clouds of digraphs in the plane *Density* × *Transitivity*. Each cloud consists of 1000 points. Each point corresponds to a 100-vertex graph produced by one of the four models being compared (note that the SF model does not allow the construction of dense graphs).

Figure 17 shows the range of transitivity values at different values of graph density for the RP, ERG, SF, and SW models. The results here differ considerably from those for reciprocity: the RP cloud not only embraces the other three clouds, but also covers a new region in the area corresponding to high-density graphs.

### 6.6. Average Clustering Coefficient

The last local property studied is the *local clustering coefficient*, which shows the extent to which the vertices of a graph form tightly connected local communities. This measure is of particular importance in the study of social networks. The clustering coefficient of a vertex is equal to the proportion of existing edges between its neighbors, out of all the possible edges between these neighbors (see [31] for the formal definition and an algorithm).

**Figure 18:**
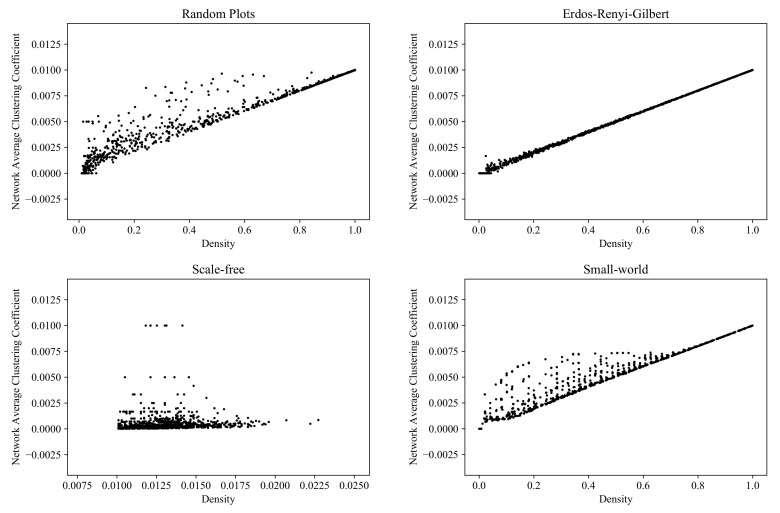
Clouds of digraphs in the plane *Density* × *Average clustering coefficient*. Each cloud consists of 1000 points. Each point corresponds to a 100-vertex graph produced by one of the four models being compared (note that the SF model does not allow the construction of dense graphs).

Figure 18 shows the distribution of the vertex-averaged local clustering coefficients for the RP, ERG, SF, and SW models (computed with the NetworkX function [56]).

### 6.7. Average Eigenvector Centrality

The final scalar property considered is *eigenvector centrality*, which measures the “importance” of each vertex in the network. Here, importance is calculated in terms of how many neighbors point to this vertex and how important these neighbors are by themselves (see [33] for the details). One of the best-known applications of eigenvector centrality is web page scoring, which is widely used by search engines.

**Figure 19:**
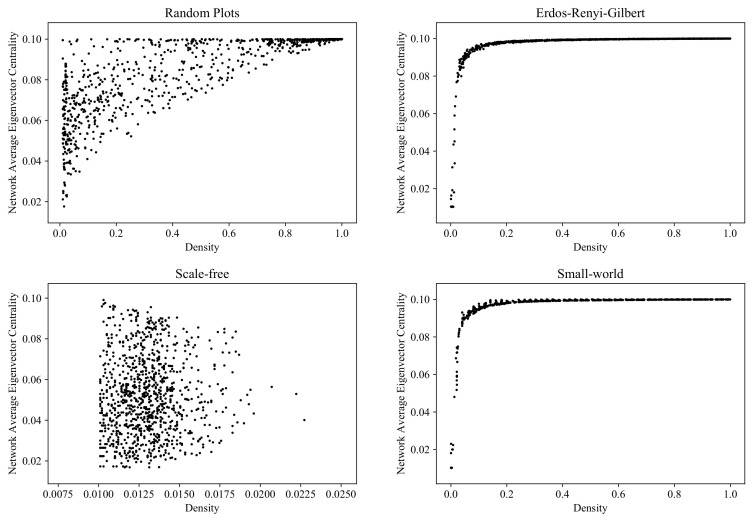
Clouds of digraphs in the plane *Density* × *Average eigenvector centrality*. Each cloud consists of 1000 points. Each point corresponds to a 100-vertex graph produced by one of the four models being compared (note that the SF model does not allow the construction of dense graphs).

Figure 19 shows the distribution of vertex-averaged eigenvector centrality values for the graphs generated by the RP, ERG, SF, and SW models (computed with the NetworkX function [57]).

### 6.8. Rumor Epidemic

The analysis of the diversity of static graph properties is not the only way to characterize the diversity of the graphs generated by a random graph model. Another indicator of the diversifying power of the model is the variety of states that a certain dynamic process takes in the graphs generated by the model. Choosing an overly restrictive model for a random graph may lead to a wrong conclusion of the form “the process always ends with YYY” or at least “the final state ZZZ never occurs”. Such narrowing conclusions may form a dangerous “false calm” opinion in many sensitive applications, one of which is epidemic modeling. Here, we consider an epidemic of rumors, where, in contrast to an epidemic of disease, each node can be both “spoiled” (with a rumor) and “cured” (with a contradiction) by each of its neighbors. We expect such a two-way process to be more sensitive to the structure of underlying graphs compared to the one-way dissemination of infection.

In our experiments, we generated 100 random graphs with 300 nodes for each of the 4 random graph models (RP, ERG, SF, SW) and each of the 5 graph density bins: $DB_{1}=\left[0,0.2\right),DB_{2}=\left[0.2,0.4\right),DB_{3}=\left[0.4,0.6\right),DB_{4}=\left[0.6,0.8\right),DB_{5}=\left[0.8,1\right]$. For each of the 100·4·5 generated graphs, we repeated the following 10 times:

(1) Infect $N_{0}$% of nodes with a rumor.

(2) Repeat the rumor-spreading process for 100 iterations. At each iteration, each spoiled node can infect each of its healthy neighbors with a probability of $p_{1}$, and each healthy node can cure each of its spoiled neighbors with a probability of $p_{2}$. The time is discrete, so the nodes do not change their state until the end of the iteration, e.g., if a healthy node is infected in the current iteration, it continues to act as a healthy node until the next iteration.

(3) At the end of the 100th iteration, capture one of the three possible states: all 300 nodes are spoiled; all are healthy; mixed—some spoiled, some healthy.

Thus, for each combination of values of the 5 input parameters (graph model, density quintile, $N_{0},p_{1},p_{2}$) we collected 1000 terminal states (100 graphs × 10 repetitions). In the computational experiments, we explored $N_{0}\in \left\{1\%,5\%,10\%\right\}$ and $p_{1},p_{2}\in \left\{0.2,0.4,0.6,0.8,1\right\}$. The experiments showed that:

(1) The terminal state “all spoiled” was never observed.

(2) In $DB_{1}$, all four random graph models showed some “all healed” and some “mixed” terminal states among the 1000 repetitions for all combinations of the other input parameters.

(3) For the graph models ERG, SF, and SW, the terminal state “mixed” was never observed in $DB_{2}$–$DB_{5}$.

(4) For the RP graph model in $DB_{2}$, both “all healthy” and “mixed” terminal states were observed for all combinations of $N_{0}$, $p_{1}$, and $p_{2}$, except for the single combination $N_{0}=1\%$, $p_{1}=0.8$, $p_{2}=0.4$, where all 1000 experiments ended in the “all healthy” state.

(5) For the RP graph model in $DB_{3}$, in 42 experiments out of 75, both “all healthy” and “mixed” terminal states were observed, and the remaining 33 experiments ended in the “all healthy” state.

(6) In $DB_{4},DB_{5}$ all experiments with the RP graph model ended in the “all healthy” state.

By relying on the graph models ER, SF, or SW, the obtained results could suggest that the rumor epidemic ceases by the 100th iteration in all graphs, except for rather sparse ones; however, the RP model revealed many graphs with an average density, for which the epidemic was not over by the 100th iteration (see Table 1).

## 7. Conclusions

In this paper, we proposed a method, which we call the Random Plots method, for constructing diverse sets of digraphs. These sets may be of practical use for modeling complex systems in which the structure of the connections between elements is not understood (either because this information is too difficult or expensive to obtain, or because it is not available in principle). In this situation, instead of trying to guess the unknown connection structure or averaging it by the maximum-entropy graph generation model, one can study a set of graphs covering “all sensible connection structures”.

The Random Plots method starts with the construction of a diverse set of pairs of piecewise linear nondecreasing plots, which is used as a reference vertex for in- and outdegree plots. At the second step, a digraph whose actual vertex in- and outdegree plots are similar to the reference plot is constructed.

To test the effectiveness of our approach, we compared the diversity of the graph sets produced by our model to that of the sets produced by some well-known graph generation models (the Erdős–Rényi–Gilbert, scale-free, and small-world models). To make the notion of “diversity” concrete, we studied each set with respect to several different scalar properties of graphs. Each graph gave rise to a point in a 2D space of the form *Density × Scalar property*, so we were able to understand the diversity of each graph set in terms of the given scalar property via the corresponding scatter of points in the plane. Our experiments showed that for 6 out of the 7 scalar properties considered, the Random Plots approach produced quite diverse clouds that not only embraced the clouds corresponding to the Erdős-Rényi-Gilbert, scale-free, and small-world models, but also covered some new areas in the 2D parameter spaces.

Another characteristic used to assess the diversity of graph ensembles was the variety of terminal states of a dynamic process—the rumor epidemic. Our experiments showed that for the graphs generated by the Erdős–Rényi–Gilbert, scale-free, and small-world models, the epidemic stopped by the end of the observation period in all cases, except for in sparse graphs. The proposed Random Plots model additionally revealed graphs of medium density for which the epidemic did not stop during the observation period. These results suggest that the Random Plots model may help, for example, to avoid dangerous “false calm” errors in the modeling of an epidemic in societies where the connection structure has been weakly studied.

The proposed approach could be improved in two ways: (1) by increasing the expressive power of Algorithm 1 (aimed, for example, at enhancing its ability to generate various SF-type graphs) and (2) reducing the time complexity of Algorithm 2. The first problem could be addressed by introducing more sophisticated rVDPs, for example, piecewise linear functions. To reduce the time complexity of graph construction, we propose that either the method of graph synthesis could be simplified (with a corresponding loss in accuracy) or we could proceed from simple graphs to multigraphs (which are considerably easier to generate). Further research could also be devoted to introducing a proper probability space for sample spaces consisting of random plots.

## Figures and Tables

**Table 1:** The percentages of “mixed” terminal states (some vertices are infected, some are healthy) of the rumor epidemic process shown by different random graph models for different graph densities.

Density Bin	Random Plots	Scale-Word	Scale-Free	Erdős–Rényi–Gilbert
1–[0,0.2)	4.33	7.03	88.87	6.33
2–[0.2,0.4)	1.06	0	0	0
3–[0.4,0.6)	0.13	0	0	0
4–[0.6,0.8)	0	0	0	0
5–[0.8,1]	0	0	0	0

## Data Availability

Not applicable.

## Appendix A

**Table A1:** Examples of VDPs for real-world graphs (OY—vertex degree; OX—vertex counting number in ascending order of degrees). Data were taken from the Index of Complex Networks (ICON) [58]. Blue and red correspond to in- and outdegrees in directed graphs, green corresponds to degrees in undirected graphs.

	Description	|V|	|E|	Vertex Degree Plots	Possible rVDPs
[31]	Nematode *Caenorhabditis elegans* neural network graph. The neurons are the vertices and the synaptic connections are the edges (the authors suggest that this is a small-world graph).	297	2359		
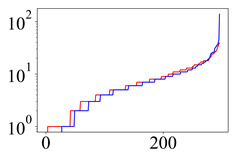
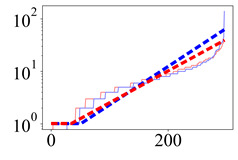
[59]	DBLP citation database. The vertices are the articles and the edges are the citations (no information about the type of vertex degree distribution).	12590	49,759		
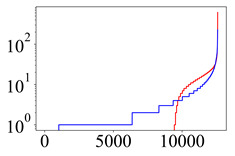
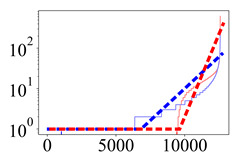
[60]	World Wide Web “darknet” segment. The vertices are the domains and the edges are the hyperlinks (the authors suggest that this is a scale-free graph).	7178	25,104		
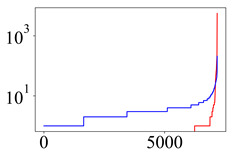
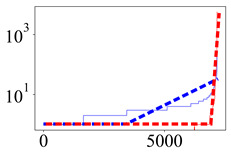
[61]	Marvel Universe graph. The vertices are the characters and an edge exists if two characters appear in a story together (surprisingly this graph does not resemble a real-world social network).	19428	96,662		
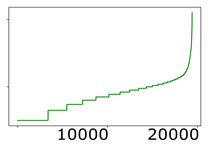
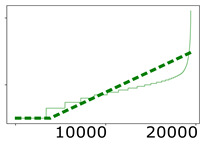
[62]	Graph showing the influence of programming languages on each other (the authors note that this graph has a power-law indegree distribution and an exponential outdegree distribution).	352	741		
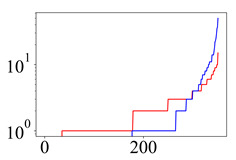
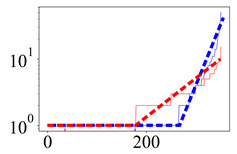
[63]	Graph showing the physical connections of the cells in a stem of *Arabidopsis Columbia* (no information about the type of vertex degree distribution).	2210	12,188		
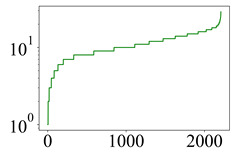
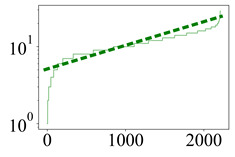
[64]	Rome road network (1999). The vertices are the crossroads (no information about the type of vertex degree distribution).	3353	8859		

## References

[1] Bonato A., López-Ortiz A., Hamel A.M. (2005). A survey of models of the web graph. Combinatorial and Algorithmic Aspects of Networking.

[2] Enikeev R. (2011). Internet Map. http://internet-map.net.

[3] Backstrom L., Huttenlocher D., Kleinberg J., Lan X. Group formation in large social networks: Membership, growth, and evolution. Proceedings of the 12th ACM SIGKDD International Conference on Knowledge Discovery and Data Mining, KDD ’06, Association for Computing Machinery.

[4] Cha M., Haddadi H., Benevenuto F., Gummadi K. Measuring user influence in twitter: The million follower fallacy. Proceedings of the 4th International AAAI Conference on Weblogs and Social Media (ICWSM).

[5] Sporns O. (2013). Structure and function of complex brain networks. Dialogues Clin. Neurosci..

[6] Sporns O. (2018). Graph theory methods: Applications in brain networks. Dialogues Clin. Neurosci..

[7] Hashimoto A., Nagao A., Okuda S. (2018). Topological graph description of multicellular dynamics based on vertex model. J. Theor. Biol..

[8] Jackson M., Duran-Nebreda S., Bassel G. (2017). Network-based approaches to quantify multicellular development. J. R. Soc. Interface.

[9] Szklarczyk D., Gable A., Lyon D., Junge A., Wyder S., Huerta-Cepas J., Simonovic M., Doncheva N., Morris J., Bork P. (2019). STRING v11: Protein-protein association networks with increased coverage, supporting functional discovery in genome-wide experimental datasets. Nucleic Acids Res..

[10] Broido A.D., Clauset A. (2019). Scale-free networks are rare. Nat. Commun..

[11] Holme P. (2019). Rare and everywhere: Perspectives on scale-free networks. Nat. Commun..

[12] Li L., Alderson D., Willinger W., Doyle J. (2004). A First-Principles Approach to Understanding the Internet’s Router-Level Topology.

[13] Tanaka R. (2005). Scale-rich metabolic networks. Phys. Rev. Lett..

[14] Tanaka R., Yi T.-M., Doyle J. (2005). Some protein interaction data do not exhibit power law statistics. FEBS Lett..

[15] Willinger W., Alderson D., Doyle J. (2009). Mathematics and the Internet: A Source of Enormous Confusion and Great Potential. Not. Am. Math. Soc..

[16] Cleland C.E. (2019). Moving beyond definitions in the search for extraterrestrial life. Astrobiology.

[17] Shahar A., Driscoll P., Weinberger A., Cody G. (2019). What makes a planet habitable?. Science.

[18] Voitalov I., Hoorn P.v., Kitsak M., Papadopoulos F., Krioukov D. (2020). Weighted hypersoft configuration model. Phys. Rev. Res..

[19] Erdös P., Rényi A. (1959). On Random Graphs I. Publ. Math. Debr..

[20] Gilbert E.N. (1959). Random graphs. Ann. Math. Stat..

[21] Bollobás B., Borgs C., Chayes J., Riordan O. Directed scale-free graphs. Proceedings of the Fourteenth Annual ACM-SIAM Symposium on Discrete Algorithms, SODA ’03, Society for Industrial and Applied Mathematics.

[22] Kumar R., Raghavan P., Rajagopalan S., Sivakumar D., Tomkins A., Upfal E. Stochastic models for the web graph. Proceedings of the 41st Annual Symposium on Foundations of Computer Science.

[23] Pachon A., Sacerdote L., Yang S. (2018). Scale-free behavior of networks with the copresence of preferential and uniform attachment rules. Phys. Nonlinear Phenom..

[24] Albert R., Barabasi A.-L. (2002). Statistical mechanics of complex networks. Rev. Mod. Phys..

[25] Faloutsos M., Faloutsos P., Faloutsos C. On Power-Law Relationships of the Internet Topology. Proceedings of the Conference on Applications, Technologies, Architectures, and Protocols for Computer Communication, SIGCOMM ’99, Association for Computing Machinery.

[26] Barabási A.-L., Albert R. (1999). Emergence of scaling in random networks. Science.

[27] Kim H.J., Kim I.m., Lee Y. (2002). Scale-free network in stock markets. J. Korean Phys. Soc..

[28] Jeong H., Mason S.P., Barabási A.-L., Oltvai Z.N. (2001). Lethality and centrality in protein networks. Nature.

[29] Toivonen R., Onnela J.-P., Saramäki J., Hyvönen J., Kaski K. (2006). A model for social networks. Phys. A Stat. Mech. Its Appl..

[30] Zanette D.H. (2002). Dynamics of rumor propagation on small-world networks. Phys. Rev. E.

[31] Watts D.J., Strogatz S.H. (1998). Collective dynamics of ’small-world’ networks. Nature.

[32] Goodfellow I.J., Pouget-Abadie J., Mirza M., Xu B., Warde-Farley D., Ozair S., Courville A., Bengio Y. (2014). Generative adversarial nets. Proceedings of the 27th International Conference on Neural Information Processing Systems, NIPS’14.

[33] Newman M. (2010). Networks: An Introduction.

[34] Xiao B., Hancock E.R., Yeung D.-Y., Kwok J.T., Fred A., Roli F., de Ridder D. (2006). A spectral generative model for graph structure. Structural, Syntactic, and Statistical Pattern Recognition.

[35] Molloy M., Reed B. (1998). The size of the giant component of a random graph with a given degree sequence. Comb. Probab. Comput..

[36] Park J., Newman M.E.J. (2004). Statistical mechanics of networks. Phys. Rev. E.

[37] Bianconi G. (2007). The entropy of randomized network ensembles. EPL (Europhys. Lett.).

[38] Kesavan H.K. (2009). Jaynes’ Maximum Entropy Principle.

[39] Erdős P., Rényi A. (1963). Asymmetric graphs. Acta Math. Acad. Sci. Hung..

[40] Wormald N.C. (1987). Generating random unlabelled graphs. SIAM J. Comput..

[41] Eggleton R.B., Hajnal A. (1975). Graphic sequences and graphic polynomials. Infinite and Finite Sets.

[42] Blitzstein J., Diaconis P. (2010). A sequential importance sampling algorithm for generating random graphs with prescribed degrees. Internet Math..

[43] Bayati M., Kim J.H., Saberi A. (2010). A sequential algorithm for generating random graphs. Algorithmica.

[44] Britton T., Deijfen M., Martin-Löf A. (2006). Generating simple random graphs with prescribed degree distribution. J. Stat. Phys..

[45] Chung F., Lu L. (2002). The average distances in random graphs with given expected degrees. Proc. Natl. Acad. Sci. USA.

[46] Chung F., Lu L. (2002). Connected components in random graphs with given expected degree sequences. Ann. Comb..

[47] Molloy M., Reed B. (1995). A critical point for random graphs with a given degree sequence. Random Struct. Algorithms.

[48] (2019). NetworkX 2.4, Erdös-Rényi Graph Generator. https://networkx.github.io/documentation/stable/reference/generated/networkx.generators.random_graphs.erdos_renyi_graph.html.

[49] (2019). NetworkX 2.4, Scale-Free Graph Generator. https://networkx.github.io/documentation/stable/reference/generated/networkx.generators.directed.scale_free_graph.html.

[50] Song F. (2014). A Super-Simple Way to Generate Directed and Undirected Watts-Strogatz Small-World Networks. http://www.nervouscomputer.com/hfs/super-simple-watts-strogatz/.

[51] Chernoskutov M., Ivanko E. (2020). Random Plots–Random Graph Generator. https://github.com/imm-complexity-lab/random_graph_gen.

[52] (2019). NetworkX 2.4, Strongly Connected Components. https://networkx.github.io/documentation/stable/reference/algorithms/generated/networkx.algorithms.components.strongly_connected_components.html.

[53] Estrada E., Rodriguez-Velazquez J.A. (2005). Spectral measures of bipartivity in complex networks. Phys. Rev. Stat. Nonlinear Soft Matter Phys..

[54] (2019). NetworkX 2.4, Spectral Bipartivity. https://networkx.github.io/documentation/stable/reference/algorithms/generated/networkx.algorithms.bipartite.spectral.spectral_bipartivity.html.

[55] Song H.F., Wang X.-J. (2014). Simple, distance-dependent formulation of the Watts-Strogatz model for directed and undirected small-world networks. Phys. Rev. E.

[56] (2019). NetworkX 2.4, Average Clustering Coefficient. https://networkx.github.io/documentation/stable/reference/algorithms/generated/networkx.algorithms.cluster.average_clustering.html.

[57] (2019). NetworkX 2.4, Eigenvector Centrality. https://networkx.github.io/documentation/stable/reference/algorithms/generated/networkx.algorithms.centrality.eigenvector_centrality.html.

[58] Clauset E.T.A., Sainz M. (2016). The Colorado Index of Complex Networks. https://icon.colorado.edu/.

[59] Ley M. (2002). The dblp computer science bibliography: Evolution, research issues, perspectives. Proceedings of the 9th International Symposium on String Processing and Information Retrieval, SPIRE.

[60] Griffith V., Xu Y., Ratti C. (2017). Graph theoretic properties of the darkweb. arXiv.

[61] Alberich R., Miró-Julià J., Rosselló F. (2002). Marvel Universe Looks Almost Like a Real Social Network. arXiv.

[62] Valverde S., Sole R. (2015). Punctuated equilibrium in the large-scale evolution of programming languages. J. R. Soc. Interface R. Soc..

[63] Jackson M.D., Xu H., Duran-Nebreda S., Stamm P., Bassel G.W. (2017). Topological analysis of multicellular complexity in the plant hypocotyl. Elife.

[64] (2016). 9th DIMACS Implementation Challenge-Shortest Paths. http://users.diag.uniroma1.it/challenge9/.

